# Socio-demographic factors and edentulism: the Nigerian experience

**DOI:** 10.1186/1472-6831-4-3

**Published:** 2004-11-22

**Authors:** Temitope Ayodeji Esan, Adeyemi Oluniyi Olusile, Patricia Adetokunbo Akeredolu, Ayodeji Omobolanle Esan

**Affiliations:** 1 Department of Restorative Dentistry, Obafemi Awolowo University, Ile-Ife, Nigeria; 2 Department of Restorative Dentistry, Obafemi Awolowo University, Ile-Ife, Nigeria; 3 Department of Restorative Dentistry, University of Lagos, Lagos, Nigeria; 4 Department of Preventive Dentistry, Obafemi Awolowo University Teaching Hospitals complex, Ile-Ife, Nigeria

## Abstract

**Background:**

The rate of total edentulism is said to be increasing in developing countries and this had been attributed mainly to the high prevalence of periodontal diseases and caries. Several reports have shown that non-disease factors such as attitude, behavior, dental attendance, characteristics of health care systems and socio-demographic factors play important roles in the aetiopathogenesis of edentulism. The aim of this study was to assess the relationship between socio-demographic factors and edentulism.

**Methods:**

A total of 152 patients made up of 80 (52.6%) males and 72 (47.4%) females who presented in two prosthetic clinics located in an urban and a rural area were included in the study. The relationship between gender, age, socio-economic status and edentulism in this study population was established.

**Results:**

No significant relationship between gender and denture demand was noted in the study. The demand for complete dentures increased with age while the demand for removable partial dentures also increased with age until the 3^rd ^decade and then started to decline. A significant relationship was found between denture demand and the level of education with a higher demand in lower educational groups (p < 0.001). In addition, the lower socio-economic group had a higher demand more for prostheses than the higher group.

**Conclusions:**

The findings in this study revealed a significant relationship between socio-demographic variables and edentulism with age, educational level and socio-economic status playing vital roles in edentulism and denture demand.

## Background

Edentulism (partial or total) is an indicator of the oral health of a population [1]. It may also be a reflection of the success or otherwise of various preventive and treatment modalities put in place by the health care delivery system [2]. Many patients also regard edentulism as self-mutilating and may be a strong incentive to seek dental treatment [3].

While the rate of total edentulism is decreasing in developed countries, the reverse is the case with developing countries and this had been attributed mainly to the high prevalence of periodontal diseases and caries [5-7].

Previous studies have also shown that several non-disease factors such as attitude, behavior, dental attendance, characteristics of health care system and socio-demographic factors play important roles in the aetiopathogenesis of edentulism [3].

Some studies reported that the incidence of edentulism correlated with educational levels and income status, with those in the lower levels exhibiting higher risks of becoming totally edentulous [8,9]. In addition, a study done in a rural area of Eastern Guatemala showed that social and environmental influence such as poverty, lack of proper education and inadequate diet contributed to widespread premature and heavy losses of permanent teeth [10].

Although, Hoover and McDermount [11] reported a higher prevalence of edentulism in males than females, Marcus et al observed that the prevalence of edentulism had no relationship with gender [12]. They also observed that there was an inverse relationship between the level of education, income and edentulism.

Studies among Nigerians have linked some of these socio-demographic factors with the prevalence, pattern and rate of dental diseases [13,14] but there has been no report on the influence of these on edentulism.

The aim of this study therefore was to assess the relationship between socio-demographic variables with types of edentulism.

## Methods

All patients that attended and were treated in the removable prosthetic units of Obafemi Awolowo University Teaching Hospitals Complex (OAUTHC), Ile-Ife (a rural area located in the south west of Nigeria) between the months of March and May year 2002 and Lagos University Teaching Hospitals (LUTH), Lagos (an urban area also located in south west Nigeria) between December 2002 and March 2003 were included in the study. Information such as age, gender, occupation and level of education attained were documented. The types of partial denture received following treatment at the clinics were also documented

There has not been a consensus on various socio-economic classifications in Nigeria because of the unstructured nature of the society. Therefore, for the purpose of this study, a standard occupational classification system designed by Office of population Census and Surveys, London (OPCS 1991) [15] modified based on local reality was used and patients were classified into three socio-economic groups:

Class 1 = Skilled worker e.g professionals and managerial officers and retirees of this cadre.

Class 2 = Unskilled workers e.g. Artisans and traders

Class 3 = Dependants. e.g. Retirees of class 2, those not on pensions, house wives of class 2 cadre, students whose parents are unskilled workers

Data was analysed using SPSS for Windows version 10.0, (SPSS Inc Chicago Illinois, USA). Analysis included frequency, cross tabulations, calculation of means. Association between discrete variables was tested by Chi-Square and the level of significance was set at 5%.

## Results

One hundred and fifty two patients attended the prosthetic clinics during the study period. Eighty (52.6%) were males while 72 (47.4%) were females (Table 1). Their ages ranged from 8 to 84 years. The median age was 22.00 years, while the mean age was 41.8 (±19.5) years. The mean age for Ile-Ife study population was 41.3 (±20.46) years, while that of Lagos was 39.9 (±17.56) years.

**Table 1:** Distribution by gender.

Gender	Ile-Ife		Lagos		Total	
	
	No	%	No	%	No	%
Male	48	48.0	32	61.5	80	52.6
Female	52	52.0	20	38.5	72	47.4
Total	100	100.0	52	100.0	152	100.0

χ^2 ^= 2.515, df = 1, P = 0.113.

There were no statistically significant age (p = 0.312) and gender (p = 0.113) differences between the populations from the two centers. (Tables 1 and 2).

**Table 2:** Denture demand by age and center/clinic

**Age group**	**Ile-Ife**		**Lagos**		**Total**	
	
	N	%	N	%	N	%
≤20	18	18.0	5	9.6	23	15.13
21–40	35	35.0	22	42.3	57	37.5
41–60	25	25.0	17	32.7	42	27.63
≥61	22	22.0	8	15.4	30	19.74
**Total**	**100**	**100.0**	**52**	**100.0**	**152**	**100.00**

χ^2 ^= 3.568, df = 3, P = 0.312

**Figure 1:**
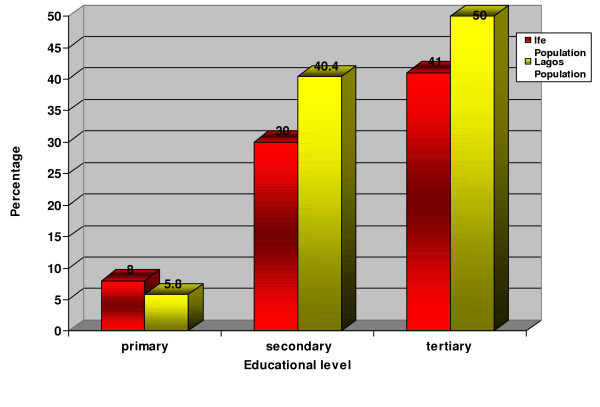
Distribution of patients according to educational level.

There was a highly significant difference in the educational status of patients seen at LUTH and OAUTHC with patients seen at LUTH being of higher educational levels than patients seen at OAUTHC. (χ^2 ^= 7.50 df = 3, P < 0.001) (Figure 1).

**Figure 2:**
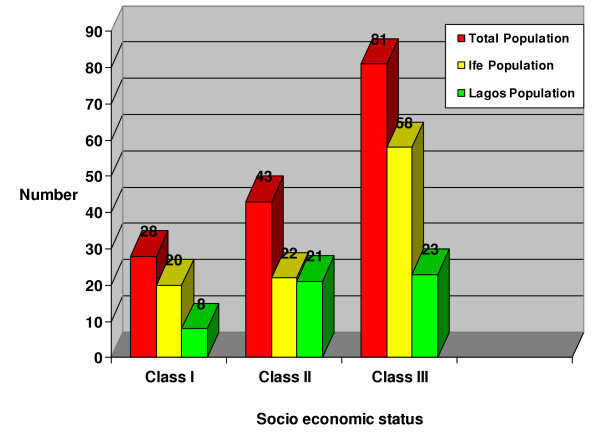
Socio-economic status distribution.

In terms of socioeconomic status, 28 (18.42%) patients belonged to class I; 43(28.29%) patients belonged to class II while 81(53.29%) belonged to class III. There was no statistically significant difference in the socio-economic status of patients from the two centers ((χ^2 ^= 5.70, df = 2, p = 0.057). (Figure 2)

In both centers, 134 patients (88.2%) received removable partial dentures, 13 patients (8.6%) received complete dentures while 5 patients (3.3%) received either upper or lower complete dentures. There was no significant difference in the demand for different types of dentures between the study locations. (P = 0.315). (Table 3).

**Table 3:** Demand for various types of denture by study location.

**Types of dentures**	**Ile-Ife**		**Lagos**		**Total**	
	
	N	%	N	%	N	%
Complete	11	11.0	2	3.8	13	8.6
Lower or upper complete denture	3	3.0	2	3.8	5	3.3
Removable partial denture	86	86.0	48	92.3	134	88.2
**Total**	**100**	**100.0**	**52**	**100.0**	**152**	**100.0**

χ^2 ^= 3.568, df = 3, P = 0.312

For the purpose of analysis, complete and lower/upper complete denture columns were merged.

However, there was a significantly higher demand for removable partial dentures than any other type of prostheses. (P < 0.01). (Table 3)

It was observed that as the age increased, the proportions demanding for complete dentures also increased. In addition, those in age group 21–40 years demanded more for removable partial denture than any other age groups. While those above 61 years asked more for removable complete dentures than removable partial dentures. (Table 4).

**Table 4:** Types of denture demand within each age group.

**Age group**	**Denture demanded**				**TOTAL**
	
	**Complete**		**Partial**		
		
	**no**	**%**	**no**	**%**	**no**
≤20	1	4.3	22	95.7	23
21–40	2	3.5	55	96.5	57
41–60	5	11.9	37	88.1	42
≥61	10	33.3	20	66.7	30
**Total**	**18**	**11.8**	**134**	**88.2**	**152**

Likelihood-ratio χ^2 ^= 16.579, df = 3, P = 0.001

**Figure 3:**
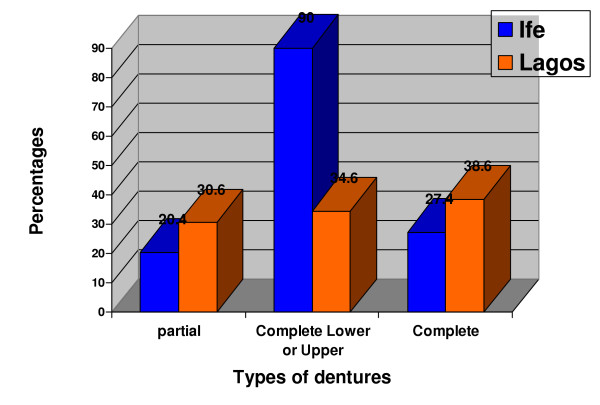
Types of prostheses demanded by centers

No significant relationship between gender and pattern of denture demand (p = 0.812) was noted and no statistically significant difference was noted in the pattern of denture demand between the two centers (p = 0.277). (Table 5 and Figure 3).

**Table 5:** Demand for various prostheses in relation to gender.

**Type of Denture**	**male**	**Female**	**Total**
Complete denture	6	7	13
Complete upper or Lower denture	3	2	5
Partial denture	71	63	134
**Total**	**80**	**72**	**152**

χ^2 ^= 0.57, df = 1, p = 0.812

For the purpose of analysis, complete and lower/upper complete denture columns were merged.

The lower educational groups demanded more for complete dentures among those asking for complete denture, while those with higher level of education asked more for removable partial denture. (P < 0.001). (Table 6). More over, those with tertiary level of education constituted the majority of the study population. (Table 6)

**Table 6:** Demand for dentures according to educational level

**Educational Level**	**Complete Denture**		**Partial Denture**		**Total**	
	
	**No**	**%**	**No**	**%**	**No**	**%**
**Nil**	11	61.1	12	8.9	23	15.1
**Primary**	3	16.7	8	5.9	11	7.2
**Secondary**	0	0.0	51	38.1	51	33.6
**Tertiary**	4	22.2	63	47.0	67	44.1
**TOTAL**	**18**	**100.0**	**134**	**100.0**	**152**	**100.0**

Fishers exact test P < 0.001

For the purpose of analysis, secondary and tertiary educational levels' rows were merged. Also complete denture and either upper or lower complete denture column were merged

Among the patients that were completely edentulous, there was no significant difference in the demand for complete dentures between those with lower educational status and those with higher educational status. P = 0.276 (Table 7)

**Table 7:** Relationship between age group, educational level and completely edentulous state

**Age group**	**Educational Level**		
	
	**Nil**	**Primary**	**Secondary/Tertiary**
≤20	1(9.1%)	-	-
21–40	2(18.2%)	-	-
41–60	4(33.4%)	1(33.3%)	
>60	4(33.4%)	2(66.7%)	4(100%)

Likelihood-ratio χ^2 ^= 7.515, df = 6 P = 0.276

It was noted that the lower the socio-economic status the higher the demand for dentures. This picture was independent of rural or urban dwelling. (Tables 8 and 9). However, 28.3 % of those in Class II who need dentures asked for complete as opposed to 3.6% in Class I and 8.6% of those in Class III.

**Table 8:** Relationship between edentulous state and socio-economic status.

Socio-economic status	Edentulous state				Total	
		
	Partial		complete			
	
	No	%	No	%	No	%
Class I	27	96.4	1	3.6	28	18.4
Class II	33	76.7	10	23.3	43	28.3
Class III	74	91.4	7	8.6	81	53.3
Total	134	88.2	18	11.8	152	100

χ^2 ^= 7.992, df = 2, P = 0.018

**Table 9:** Socio-economic status distribution by centers

**Socio-economic Class**	**Lagos center**		**Ife center**		**TOTAL**	
	
	**NO**	**%**	**NO**	**%**	**No**	**%**
Class I	8	15.4	20	20.0	28	18.4
Class II	21	40.4	22	22.0	43	28.3
Class III	23	44.2	58	58.0	81	53.3
**TOTAL**	52	100.0	100	100.0	152	100.0

χ^2 ^= 5.70, df = 2, p = 0.057

## Discussion

Tooth loss could occur as a result of caries, periodontal diseases, trauma, tooth impaction, orthodontic reasons, hypoplasia, over eruption, supernumerary teeth, attrition, neoplastic and cystic lesions [5-7].

Many studies have consistently shown the role of specific diseases like dental caries and periodontal disease as a major cause of tooth loss [7,13,14]. This same picture was noted in similar Nigerian studies [5,6].

Okoisor further established that the disease factors responsible for tooth loss was age related; with caries and periodontal diseases being the major causes of tooth mortality in children and adult respectively [5].

However, none of the studies done in Nigeria evaluated the role of other factors such as education, socio-economic status, gender, location of patients, dental attitude and behavior in the etiology of edentulism.

The older age groups in this study required more of removable complete dentures than the younger age groups while the younger age groups required more of removable partial dentures. This is in agreement with the study done by Marcus et al [12]. Although there was an over representation of age groups >61 and 21–40 in our study population, the percentages of these age groups in Nigerian population are 4% and 30% respectively in both urban and rural areas [16]. Hence, these age groups have risk factors that might be responsible for their needing dentures.

This age related changes may not be unconnected with the deteriorative physiological changes noticed after adolescence and which gets worse with increase in age, a situation which is changing rapidly in the developed countries due to improved social infrastructure and functional health system [17,18].

Most studies have also shown significant gender difference in edentulism with more males becoming edentulous than females [11,19]. This has been attributed to the fact that males are more active than females and do not pay much attention to oral care. A significant gender difference was not seen in this study although variation in site presentation was observed. In Lagos, an urban area, more males actually demanded for prostheses. However, in Ile-Ife, a rural area more females demanded for prostheses. This is in agreement with the studies done by Eklund and Burt [8] and Marcus et al [12]. Although no statistically significant difference was noted in the rural-urban gender presentation, a larger qualitative study alongside a quantitative study may be able to adduce possible reasons for this interesting observation.

Majority of our study population belonged to the higher education status. This is because those with higher level of education are more informed about their health needs and may seek dental treatments earlier and more often than those of lower educational status who may only seek dental treatment when there is apparent morbidity. In addition, those of higher educational status are likely to be richer than those of lower educational status. Hence, they are able to afford the cost of dental treatments from time to time.

Our study showed that the need for complete dentures decreased with increasing level of education (p < 0.001), hence the likelihood of retaining teeth in the mouth becomes higher as the educational level increases. Although the educational status of the patients from the two centers differ, independent analysis of these centers still showed the same significant effect of educational status on the pattern of denture demand. This is in agreement with the findings of Brodeur et al, where the proportion of completely edentulous adults decreased from 26% in 1980 to 20 % in 1993 due to improved income and educational status [1].

The association between edentulism and educational status may be as a result of improved dental health awareness, increased utilization of oral health facilities, proper oral hygiene habits acquired during learning process and peer group influence.

Interestingly, about 23.3 % of those in class II who need dentures asked for complete dentures as opposed to 3.6% in class I and 8.6% in class III. The reason for this may be as a result of the fact that they may not be able to afford the exorbitant cost of restorative procedures hence they wait until they have lost their set of teeth to have a complete removable denture which is cheaper.

The present study showed that people with low socio-economic status demanded for more dentures than the high socio-economic group. Studies have long established a gradient relationship between socio-economic status and health [20]. In so many intricate ways, socio-economic status tends to affect health behaviors, the environment and social influences an individual is exposed to. Hunter and Arbona found that environmental influences such as land hunger, family poverty, and inadequate diet are of paramount importance in the cause of tooth loss [10]. They concluded, "Periodontal disease drives the poorest of the poor to spend disproportionately large sums on pain killers and destructive traditional medicine".

The importance of socio-economic status is further reflected in the urban-rural variance noted in this study population's demand for denture. The Ile-Ife study group, a rural population with a lower socio-economic status, demanded for more dentures than the Lagos study group.

However, this study population was an all-inclusive hospital based sample; the result may not be representative of the population at large. Hence, its use can only be limited to the study population. A randomized population based survey may be able to present a better picture among Nigerians.

This study observed that edentulism is due to a combination of various factors. Poor education, a risk factor for poverty, has been identified as a major factor in edentulism. So also is the socio-economic status of the patient. These two factors, which are non-disease factors, affect the mortality of teeth arising from disease factors. There is therefore a need for oral health policy formulators to focus on improving the educational and socio-economic status of its citizens (a down stream approach) rather than the present emphasis on disease control (an up stream approach) in oral health care delivery.

On the other hand, with increasing level of literacy, and positive social changes in Nigeria, Prosthodontists should brace up to face the challenges that may arise from increased removable partial denture demand and decreased demand for complete dentures. This is because, with increase in level of education and socio-economic status of patients, the demand for removable partial dentures is likely to increase while dentists may be confronted with a significant increase in the number of difficult edentulous mouths requiring treatment.

In addition to addressing the non-disease factors, dental education should be targeted at the uneducated populace, the rural dwellers and low-income groups to reduce the rate of total edentulism.

## Conclusion

No gender relationship with denture demand was noted this study. In addition, the demand for complete dentures increased with age. There was a statistically significant inverse relationship between educational levels and demand for dentures. There was more demand for prostheses among the lower socio economic groups.

## Pre-publication history

The pre-publication history for this paper can be accessed here:


